# Total abdominal hysterectomy in a patient with immune thrombocytopenic Purpura: A case report

**DOI:** 10.1016/j.ijscr.2023.109102

**Published:** 2023-12-06

**Authors:** Willbroad Kyejo, Gregory Ntiyakunze, Brenda Moshi, Neema Lubuva, Munawar Kaguta, Shweta Jaiswal

**Affiliations:** a Department of Family Medicine, Aga Khan University, P.O. Box 38129, Dar Es Salaam, Tanzania; b Department of Obstetrics and Gynecology, Aga Khan Hospital, P.O. Box 2289, Dar Es Salaam, Tanzania; c Department of Hematology, Aga Khan Hospital, P.O. Box 2289, Dar Es Salaam, Tanzania

**Keywords:** Immune thrombocytopenic purpura, TAH, Preoperative preparation

## Abstract

**Introduction:**

Immune Thrombocytopenic Purpura poses unique challenges in surgical settings due to an increased risk of bleeding. This report details the perioperative management of a patient with Immune Thrombocytopenic Purpura undergoing Total Abdominal Hysterectomy, emphasizing the importance of tailored approaches for such cases.

**Case presentation:**

A 41-year-old female with Immune Thrombocytopenic Purpura and symptomatic uterine fibroids, despite medical management, opted for Total Abdominal Hysterectomy. Prednisolone therapy and platelet transfusion were used preoperatively to optimize platelet counts.

**Discussion:**

Effective management was achieved through meticulous surgery, continued prednisolone therapy, and vigilant postoperative monitoring. This case highlights the value of a multidisciplinary approach in ensuring positive surgical outcomes for Immune Thrombocytopenic Purpura patients.

**Conclusion and recommendation:**

This case underscores the significance of individualized perioperative care for Immune Thrombocytopenic Purpura patients undergoing major surgery. By optimizing medical therapy and maintaining close monitoring, favorable results can be achieved, enhancing the quality of life for such patients. It is recommended that such comprehensive approaches are considered in similar cases.

## Highlights

• Preoperative Management: optimize the patient's platelet count before surgery.
• Surgical Considerations: Techniques used during the total abdominal hysterectomy to minimize bleeding.
• Postoperative Outcomes with Immune Thrombocytopenic Purpura.

## 1 Introduction

Immune thrombocytopenic purpura (ITP) is an autoimmune disorder characterized by the destruction of platelets, resulting in thrombocytopenia. It is often associated with an increased risk of bleeding complications. Performing surgical procedures in patients with ITP poses a unique challenge for healthcare providers, as the delicate balance between hemostasis and bleeding must be carefully managed [1]. Total abdominal hysterectomy (TAH) is a common gynecological procedure that may be necessary for various indications, such as uterine fibroids or malignancies [2]. However, performing TAH in patients with ITP requires a multidisciplinary approach and specialized perioperative management strategies to minimize bleeding risks and ensure optimal patient outcomes.

The management of ITP typically involves the use of immunosuppressive agents, such as corticosteroids, to reduce platelet destruction and increase platelet counts [4]. However, achieving adequate platelet levels preoperatively can be challenging, and additional interventions, such as platelet transfusion, may be required to ensure a safe surgical procedure. Furthermore, the timing of surgery must be carefully considered, considering the patient's platelet count and response to treatment [2].

This case report aims to highlight the perioperative management strategies employed in a patient with ITP who underwent TAH. The report emphasizes the importance of a multidisciplinary approach involving hematologists, anesthesiologists, and gynecologic surgeons to optimize patient safety and outcomes. By sharing this case, we hope to contribute to the existing knowledge base and provide insights into the challenges and considerations associated with performing TAH in patients with ITP**.** This paper has been reported in line with the SCARE 2020 criteria [3].

## 2 Case report

**Fig. 01:**
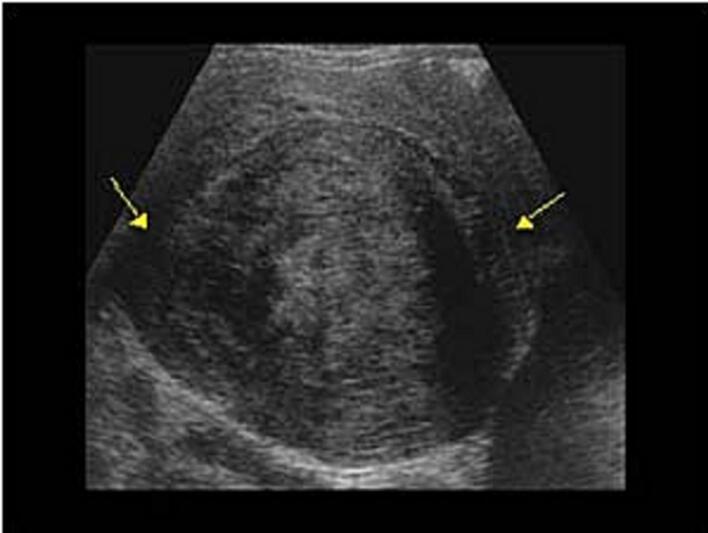
Abdominal ultrasound showing large subserosa fibroid on the dorsal aspect of the uterus.

A 44-year-old female patient presented to our gynecology clinic with symptomatic uterine fibroids. She had a known diagnosis of (ITP) for the past five years, which was managed with prednisolone therapy. Biopsy results revealed normal bone marrow. Despite the treatment, her platelet count remained persistently low at 40,000/μL. The patient had previously undergone a trial of medical management for her fibroids, including treatment with Mirena followed by Zoladex injection, but these interventions did not provide sufficient relief of her symptoms. Her initial ultrasound showed large subserosa fibroid on the dorsal aspect of uterus Fig. 01.

The patient experienced heavy and prolonged menstrual bleeding, which led to significant anemia. After a thorough discussion of the potential risks and benefits of different treatment options, including the limitations of medical management in her case, the patient made an informed decision to proceed with (TAH). The rationale behind the decision was to alleviate her symptoms, improve her quality of life, and address the underlying cause of her persistent anemia.

Preoperative assessment involved a collaborative effort between the hematologists, anesthesiologists, and gynecologic surgeons. The patient's medical history, including previous bleeding episodes and response to prednisolone therapy, was reviewed in detail. Laboratory investigations confirmed the low platelet count of **40,000/**μL, and coagulation parameters were within normal limits. A multidisciplinary team discussion concluded that preoperative optimization of prednisolone therapy, platelet transfusion, and meticulous perioperative management were crucial to minimize bleeding risks during the surgical procedure.

The patient's prednisolone therapy was optimized preoperatively, with the dose increased to 60 mg daily to suppress the immune response and improve platelet production. Platelet transfusion was planned two days prior to surgery to increase the platelet count to a safer range. The decision to perform platelet transfusion was based on the patient's clinical symptoms, platelet count, and the anticipated surgical bleeding during TAH. The transfusion aimed to raise the platelet count to a level above 150,000/μL to minimize the risk of excessive bleeding intraoperatively.

Full blood picture preoperative was as follows: White Cell count 12.1 × 10^9^ /l, Hemoglobin 10.1 g/dl, **platelets 209 × 10**^**9**^ **/l**, MCV 73.6 fl, MCHC 30.5 g/l.

During open TAH, a meticulous surgical technique was employed, with an emphasis on hemostasis. Electrocautery was used cautiously, and precise suture ligation was performed to control bleeding from vascular structures. Tranexamic acid was administered intravenously as a prophylactic measure to further reduce bleeding during the surgery. The surgical team remained vigilant for any signs of bleeding or hematoma formation throughout the procedure.

Postoperatively, the patient was closely monitored in the intensive care unit for the first 24 h. Platelet counts were checked daily, and additional platelet transfusions were performed as needed to maintain a safe platelet level. Continuous monitoring of vital signs, hematologic parameters, and signs of bleeding or thrombotic events was conducted. The patient's postoperative course was uneventful, with no significant bleeding complications or thrombotic events observed.

On day 7, the patient was evaluated by both the gynecologist and hematologist. The gynecologist performed a thorough examination and confirmed the absence of any surgical complications. The patient reported minimal pain and good overall recovery. The hematologist assessed the patient's platelet count, which was **platelets 145 × 10**^**9**^ **/l**. There were no signs of bleeding or thrombotic events. Based on the joint evaluation, it was determined that the patient was progressing well.

## 3 Discussion

This case report presents the successful perioperative management of a patient with (ITP) undergoing (TAH). The delicate balance between maintaining hemostasis and preventing bleeding complications is a significant concern when performing surgical procedures in patients with ITP [1,2]. This case highlights the importance of a multidisciplinary approach and specialized perioperative management strategies in achieving optimal outcomes.

The prevalence of ITP varies globally, with estimates ranging from 1.6 to 3.9 per 100,000 individuals [1]. In Tanzania, limited data are available regarding the prevalence of ITP, making this case report particularly noteworthy as the first reported case in the country. The lack of local prevalence data underscores the need for increased awareness and diagnostic capabilities to identify patients with ITP and provide appropriate management.

Morbidity rates associated with surgical interventions in patients with ITP are a significant concern. Chang et al. (2002) reported a morbidity rate of 23 % in ITP patients undergoing various surgical procedures, with bleeding and infection being the most common complications [5]. However, our case demonstrates that with careful perioperative management, including preoperative platelet transfusion and meticulous surgical techniques, complications can be minimized.

Prognosis in patients with ITP is influenced by disease severity, response to treatment, and the presence of comorbidities. The overall 5-year survival rate for patients with ITP has been reported as 91 % [1]. Although specific data on the prognosis of ITP patients undergoing hysterectomy are limited [2]. The close collaboration between hematology and surgical teams, as exemplified in this case, is vital in optimizing outcomes and ensuring long-term success.

In resource-limited settings like Tanzania, the management of complex cases such as ITP requires a multidisciplinary approach. Strengthening collaboration between hematologists and gynecologic surgeons is crucial for delivering comprehensive care to patients with ITP undergoing surgical interventions. Additionally, increased accessibility to diagnostic tools, such as platelet count monitoring and specialized laboratory testing, is essential for timely diagnosis and appropriate management [6,7].

This case report emphasizes the significance of sharing experiences and lessons learned in managing rare conditions like ITP in resource-constrained settings. By disseminating such knowledge, healthcare providers can enhance their understanding of optimal perioperative management strategies for patients with ITP and potentially improve patient outcomes.

## 4 Conclusion

This case report highlights the successful perioperative management of a patient with ITP undergoing total abdominal hysterectomy. It emphasizes the importance of collaborative care and increased awareness and diagnostic capabilities for ITP in resource-limited settings. Further studies and case reports are needed to expand our understanding of best practices.

## Informed consent

Written informed consent was obtained from the patient for publication and any accompanying images. A copy of the written consent is available for review by the Editor-in-Chief of this journal on request.

## Ethical approval

Ethical approval has been granted from our Aga khan University, with Reference no: AKU: 0176/0J6/17.

## Funding

No funding was provided for research.

## Author contribution

W.K: Study conception, production of initial manuscript, collection of data, proofreading

G.N: Revision of the manuscript, proofreading

B.M: Revision of the manuscript, proofreading

N.L: Revision of the manuscript, proofreading

M.K: Production of initial manuscript, collection of data

S.J: Study conception, production of initial manuscript, collection of data

## Guarantor

Dr. Shweta Jaiswal

## Conflict of interest statement

No conflicts of interest.
